# Modeling and dosimetric performance evaluation of the RayStation treatment planning system

**DOI:** 10.1120/jacmp.v15i5.4787

**Published:** 2014-09-08

**Authors:** Bongile Mzenda, Koki V. Mugabe, Rick Sims, Guy Godwin, Dayan Loria

**Affiliations:** ^1^ Auckland Radiation Oncology Auckland New Zealand; ^2^ Department of Medical Physics Waikato District Health Board Hamilton New Zealand; ^3^ Radiation Oncology Queensland Gold Coast University Hospital Southport Australia

**Keywords:** dosimetric accuracy, treatment planning system, commissioning, modeling

## Abstract

The physics modeling, dose calculation accuracy and plan quality assessment of the RayStation (v3.5) treatment planning system (TPS) is presented in this study, with appropriate comparisons to the more established Pinnacle (v9.2) TPS. Modeling and validation for the Elekta MLCi and Agility beam models resulted in a good match to treatment machine‐measured data based on tolerances of 3% for in‐field and out‐of‐field regions, 10% for buildup and penumbral regions, and a gamma 2%/2 mm dose/distance acceptance criteria. TPS commissioning using a wide range of appropriately selected dosimetry equipment, and following published guidelines, established the MLC modeling and dose calculation accuracy to be within standard tolerances for all tests performed. In both homogeneous and heterogeneous mediums, central axis calculations agreed with measurements within 2% for open fields and 3% for wedged fields, and within 4% off‐axis. Treatment plan comparisons for identical clinical goals were made to Pinnacle for the following complex clinical cases: hypofractionated non‐small cell lung carcinoma, head and neck, stereotactic spine, as well as for several standard clinical cases comprising of prostate, brain, and breast plans. DVHs, target, and critical organ doses, as well as measured point doses and gamma indices, applying both local and global (Van Dyk) normalization at 2%/2 mm and 3%/3 mm (10% lower threshold) acceptance criteria for these composite plans were assessed. In addition 3DVH was used to compare the perturbed dose distributions to the TPS 3D dose distributions. For all 32 cases, the patients QA checks showed > 95% of pixels passing 3% global/3 mm gamma.

PACS numbers: 87.55kd, 87.55km, 87.55de, 87.55dk

## I. INTRODUCTION

The accuracy of the dose calculation in the treatment planning procedure is strongly influenced by the treatment planning system's beam model accuracy and requires critical validation of the dose computation engine. With the growing use of advanced treatment techniques, incorporating the use of very small highly modulated fields, such as in hypofractionated and stereotactic treatments, and the increasing use of treatment adaption techniques, the accuracy demands on planning systems and their scope to contend with advanced techniques has increased in recent years.^1^, ^2^ Presently RayStation (RaySearch Medical Laboratories AB, Stockholm, Sweden) has some advanced features to tackle these challenging and complex demands.

At present a rigorous dosimetric accuracy evaluation for the basic RayStation TPS has not been reported in the literature. For example Lim and LoSasso^(3)^ assess the commissioning of Pinnacle, Eclipse, and RayStation; however, their study has particular emphasis on the multileaf collimator (MLC) aspect of the modeling and has limited information on essential dosimetric assessment of the system. Nuver et al.^(4)^ report on their initial experience with rectal cancer IMRT treatment plans for RayStation; whilst this provides useful practical clinical information, it does not present dosimetric commissioning assessment. Sutton et al.^(5)^ present their commissioning experience of RayStation (v3.0); however, the study does not show comparisons to other planning systems for RayStation's performance to be fully appreciated. Our study aims to provide insight into the modeling process and to allow the basic dose calculation accuracy and clinical plan quality evaluation of this TPS.

In this study, the beam models for the Elekta Synergy digital accelerators (Elekta AB, Stockholm, Sweden) with MLCi and Agility collimators are presented. The MLCi head consists of 80 leaves with projected width of 1.0 cm at isocenter and can overtravel the central axis by 12.5 cm. The Agility head, on the other hand, consists of 160 leaves of 0.5 cm width at isocenter which can overtravel by 15 cm, whilst the collimators in the orthogonal direction overtravel by 12 cm. There are no backup collimators in the leaf direction for the Agility model and interdigitation is allowed. The MLC thickness is 7.5 cm for the MLCi model and 9.0 cm for the Agility model.

The following more advanced features of the TPS are outside the scope of this report: deformable registration, fallback or backup planning, multicriteria optimization (MCO), dose tracking, adaptive replanning, biological optimization, and electron Monte Carlo.

## II. MATERIALS AND METHODS

Dose evaluation tools, including some of the latest software, phantom, and dosimetry equipment (including the ArcCHECK device, 3DVH, Lucy stereotactic phantom, CIRS dynamic thorax phantom, GAFCHROMIC EBT3 film, and appropriate selection of ionization chambers) were applied in this work. Special attention was taken on the tolerances used, and these varied with increasing geometrical complexity and application of more complex algorithms or optimization as required, taking into account the inherent limitations of the collapsed cone convolution superposition (CCC)‐based calculations and, in most cases, the criteria proposed by Venselaar et al.^(6)^ was followed. Moreover, to rigorously test the performance of the models, tight tolerances were used in this work for complex geometries and several publications were consulted in devising the commissioning tests and acceptance criteria (TG 53 of Fraass et al.,^(7)^ van Dyke et al.,^(8)^ IPEM 68^(9)^). The Pinnacle (v9.2; Philips Medical Systems, Andover, MA) TPS used for comparison in this work was previously commissioned for clinical use based on similar rigorous tests and tolerances.

### A. Beam modeling and validation

Beam modeling was based on the guidance by RaySearch^(10)^ and was performed in RayPhysics, the physics modeling module of the TPS. The following issues were noted during beam modeling

- The RayPhysics computed output is convolved with the entered detector sizes, whilst the RayStation output is not convolved (RayStation refers to the treatment planning module). This is particularly important for model validation, to ensure correctly matched data are compared.
- In the current version, only rectangular, centralized fields can be imported into RayPhysics for modeling purposes, and these can either be defined by MLCs only or jaws and MLC collimated, but not both.
- Average transmission is used for MLC transmission, inter and intraleaf leakage is not accounted for separately.
- MLC modeling parameters and results are verified externally of RayPhysics (particularly if jaws and MLC collimated fields are used).
- The cutoffs in the diagonals are modeled using the beam profile correction; this also has to be verified externally of RayPhysics (individual leaf position limitations cannot be entered, only one for the whole leaf bank).

#### A.1 Modeling steps

**Figure 1:**
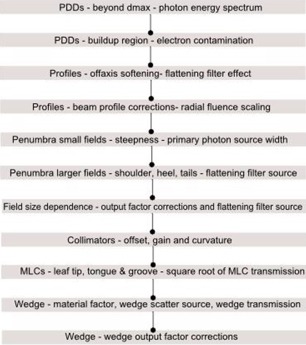
Summary of the iterative beam modeling steps followed to match the TPS beam models to the measured data.

The machine model commenced with definition of the physical characteristics. The measured data was imported and the modeling steps shown in Fig. 1 were followed. Initially the beam model normalization was set by normalizing the energy spectrum and the output factor corrections prior to computation of all curves. Electron contamination was turned off for determining the required photon energy spectrum based on the fit to measured PDDs from the depth of maximum dose ($D_{\text{max}}$) up to 40 cm depth. Output factor corrections automodeling was required to match the absolute dose level of the measured and computed curves. The energy spectrum (parameterized) automodeling step was useful for constraining the shape of the energy spectrum. Once a good match was obtained to PDDs below $D_{\text{max}}$, the electron contamination was turned back on. The electron contamination automodeling step was then used to match the PDDs for depths from the surface to $D_{\text{max}}$. For a good match at all depths, a few iterations of the photon energy spectrum, electron modeling, and output factor corrections automodeling steps were needed.

Next, the profiles were matched by use of the off‐axis softening and beam profiles corrections steps. It was found that adding more radii close to the penumbra for the largest field size was useful in getting a good match, and manual parameter adjustments worked better than automodeling for this step. The parameters of the primary source were determined from the steepness of the profiles' penumbra. The width of the primary source was manually decreased to get a steeper penumbra and increased to broaden the penumbra, as required. To match the tails and shoulders of the profiles, the flattening filter source width and weight were manually adjusted. These parameters demonstrated field size dependence, as expected. Profile mismatches observed at the penumbra FWHM (full width half maximum) point were corrected by use of the collimator calibration parameters. The width for computed profiles was adjusted by applying an offset parameter, as required, for X jaws, Y jaws, or MLC x‐position to match this to the measured profile. After this step, the PDDs were checked again and readjusted to match.

To model the MLC parameters (tongue and groove, leaf tip width, MLC collimator calibration x‐position, and MLC x‐position offset), the dose calculations from the Beam 3D Modeling module were compared to measured data. The steps pertaining to the collimators and MLCs were done after all the other preceding modeling was completed using many small fields, and the use of sweeping fields for these steps was very useful. Beam profile correction parameters were then adjusted to model the MLC positions in the diagonals by matching the Beam 3D module‐computed profiles to the measured diagonal profiles.

It should be noted that the wedge model uses the open‐field model for its starting point, then additional wedge parameters. It is therefore advisable to finish the open‐field model prior to commencing on the wedge modeling. The default parameters were a good starting point, and the PDDs for the largest field size were matched by adjusting the wedge factor whilst, for smaller fields, adjustments to the scatter source were used.

Jaw‐ and MLC‐collimated measured data were used in RayPhysics, for field sizes ranging from 1×1 cm to 40×40 cm measured at 100 cm SSD. The (virtual) phantom size of 60.1 cm was used. The size of the phantom was chosen to adequately match to the measurement conditions and provide sufficient backscatter, as well as to position the phantom at the center of the TERMA grid voxels. The absolute dose calibration for the reference field size was specified at 10 cm depth, 100 cm SSD. It is advisable to optimize output factor corrections often during the beam modeling process. This is to allow for corrections, including factors for field size dependence and model imperfections, and to continuously adjust to give a good fit to the measured data. A resolution of 0.2 cm was used during the iterative modeling; however, the final beam models were computed on a 0.1 cm resolution. This was in order to compare the final computed and measured data at the highest resolution and avoid possible discretization uncertainties due to a low resolution dose grid. It is also worth noting that a good resolution is important for modeling electron contamination in the buildup region.

#### A.2 Model validation

Penumbra width, flatness, and symmetry are computed on the measured curves in RayPhysics, and these parameters characterize the measured data. Agreement between the computed beam models and measured data is calculated in terms of the fit quality (i.e., the root mean square (RMS) difference). The fit quality, therefore, characterizes how well the beam model matches the measured data and is computed for the buildup and falloff regions for percentage depth dose (PDD) curves, as well as the in‐field (central region within 80% of maximum dose), out‐of‐field (20% of maximum dose and below), and penumbra regions for the profiles. Validation of the goodness of fit between the beam models and measured PDDs and profiles (not used in the RayPhysics modeling) was evaluated using a 2% global (G) normalization/2 mm gamma dose difference/distance‐to‐agreement acceptance criterion for open fields (MLC only defined, as well as jaw‐ and MLC‐ defined) and also for wedged fields.

### B. Dosimetric commissioning tests

The commissioning tests summarized below were performed to verify the dose accuracy, MLC modeling, and system configuration of the RayStation computed data from the MLCi and Agility beam models (6 and 10 MV, open and wedged) compared to direct linear accelerator measurements.

#### B.1 Point dose measurements

Point doses (central axis, off‐axis, oblique, wedged central axis, and wedged off‐axis) were measured in a large water tank (Scanditronix Wellhofer GmbH, Nuremburg, Germany) at 100 cm SSD. Field sizes ranged from 1×1 cm to 40×40 cm for 6 MV and 10 MV photons. Cross‐calibrated CC13 and CC01 chambers (IBA Dosimetry GmbH, Schwarzenbruck, Germany) where used for measuring point doses for field sizes greater than 4×4 cm and less than 4×4 cm, respectively. These chambers have calibrations traceable to the National Radiation Laboratory (NZ). The edge detector diode (Sun Nuclear Corporation, Melbourne, FL) and GAFCHROMIC EBT3 film (ISP Technologies, Wayne, NJ) were used to confirm small field doses and output factors. Output factors and off‐axis checks were performed for jaw‐ and MLC‐collimated fields, as well as for MLC only defined fields. Extended (120 cm) and near (80 cm) SSD checks were done for the long narrow field (i.e., 5×40 cm), as well as 5×5 cm and 10×10 cm fields.

In addition, measurements were done in a Solid Water phantom (Gammex, Middleton, WI) for a 10×10 cm field at 100 cm SSD with various thicknesses of bolus (3 mm to 20 mm) compared to the same arrangement in the TPS. Solid water slabs in various combinations with inhomogeneities including low density (lung) and high density (bone) slabs (Gammex) were used to assess the accuracy of the TPS against measurement using a calibrated Farmer‐type chamber, PTW 30013 (PTW GmbH, Freiburg, Germany).

The performance of the collapsed cone algorithm was investigated for more complex inhomogeneous geometries by measuring point doses at the center of a 1 cm diameter tissue‐equivalent sphere inside an anthropomorphic thorax phantom (CIRS Inc., Norfolk, VA). Eight equally spaced 3×3 cm cm fields were planned to deliver a near‐homogenous dose to the insert for the 6 MV Agility beam model using both the RayStation and Pinnacle TPS. The phantom was positioned using the Elekta XVI CBCT image guidance system, and the point dose was measured using a cross‐calibrated CC01 ionization chamber (IBA Dosimetry).

#### B.2 MLC film checks

Radiochromic EBT3 film (ISP Technologies) was used to verify the accuracy of the MLC transmission, as well as tongue‐and‐groove modeling parameters due to its better spatial resolution over diode arrays. EBT3 has the added advantage that orientation dependence with respect to film side is removed, and it has been shown to have insignificant energy dependence for photon beams (Borca et al.^(11^)). Batch‐to‐batch variations were minimized by using films from the same batch. Film calibration was obtained by irradiation at different doses from 0 to 400 cGy (in steps of 10 cGy up to 50 cGy, then steps of 50 cGy up to 400 cGy). The dose‐to‐film was checked against that measured using a calibrated ionization chamber at the same depth.

Picket fence, Garden fence, strip pattern, and fingers film tests were carried out to check the MLC transmission, as well as tongue‐and‐groove modeling accuracy (test segment shapes are shown in the Results section). For the Picket Fence test, additional checks were performed with overlaps and gaps comprising errors of 1 mm and 2 mm.

#### B.3 Clinical test plans

A total of 32 pairs of clinical plans were analyzed in this study. These consisted of 3D CRT, and forward‐ and inverse‐planned IMRT, as well as VMAT plans computed in RayStation and compared to equivalently optimized Pinnacle plans for the same dataset and clinical volumes to attain the same clinical goals. Critical plan quality analysis, including 3D dose grids, organs of interest doses, and DVH comparisons, were undertaken.

The ArcCHECK 3D cylindrical phantom (Sun Nuclear), in conjunction with the SNC Patient software (v6.2.3), were used for fluence map measurement and gamma analysis in absolute dose mode applying both local (L) and global (G) or Van Dyk normalization at 2%/2 mm and 3%/3 mm (10% lower threshold and measurement uncertainty applied) acceptance criteria, whilst the CC13 ionization chamber was used for point dose verification. 3DVH (v2.2; Sun Nuclear) was used for critical DVH analysis for the standard prostate plans, as well as the complex H&N and spine plans (coplanar cases only).

##### B.3.1 Standard clinical cases

These consisted of 15 prostate plans, of which eight were planned using IMRT (nine‐field step & shoot) and the others were planned using VMAT; five forward‐planned IMRT breast plans, as well as five VMAT brain plans. 3D CRT plans for palliative spine, pelvis, and brain cases were also checked.

##### B.3.2 Complex clinical cases

These consisted of a dual‐arc VMAT planned H&N case, a stereotactic inverse‐planned (eleven‐ field step & shoot) IMRT thoracic spine, and a forward‐planned (nine‐field) noncoplanar hypofractionated upper left lobe lung case.

## III. RESULTS

### A. Beam modeling and validation

**Figure 2:**
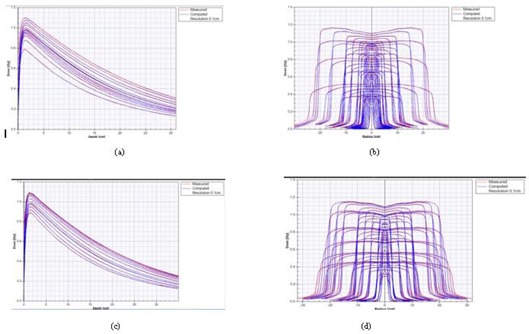
6 MV open field (a) PDDs and (b) cross‐plane profiles showing matches between measured data and RayStation for Agility beam model, and corresponding data for MLCi model (c), and (d), for field sizes from 1 × 1 cm to 40 × 40 cm (red=measured,blue=RayPhysics computed, all at resolution of 0.1 cm).

The final Agility and MLCi beam models for both open and wedged fields resulted in a good match to the measured data fed into the RayPhysics module. Figure 2 shows the match obtained for the 6 MV open photon beams for both models (from the RayPhysics modeling section), and Table 1 summarizes this data, generally showing good agreement to the measured data.

Agreement for all results was within the 2% tolerance in the fall‐off region and within the 10% tolerance in the penumbra region, and the majority of results in the buildup, in‐field, and out‐of‐field were also within 10%, 3%, and 3% tolerances, respectively. In a few instances, the tolerance values were exceeded, particularly for the in‐field (shoulder) for fields smaller than 5×5 cm and in out‐of‐field (tails) regions for field sizes greater than 15 × 15 cm. Also, in the buildup region, a few cases exceeded 10%, particularly for the larger fields in the Agility model and small fields in the MLCi model.

**Table 1:** Summary of average percentage difference and standard deviation (± 2 SD) from curve quality metrics for (a) PDDs, (b) cross‐plane profiles, and (c) in‐plane profiles (averaged over all depths (i.e., $D_{\text{max}}$, 5, 10, 15, 20, and 25 cm deep)) for square field sizes from 1 × 1 cm to 40 × 40 cm, all at SSD of 100 cm

(a)	*Agility 6 MV*		*Agility 10 MV*		*MLCi 6 MV*		*MLCi 10 MV*	
	*Buildup (%)*	*Fall‐off (%)*	*Buildup (%)*	*Fall‐off (%)*	*Buildup (%)*	*Fall‐off (%)*	*Buildup (%)*	*Fall‐off (%)*
field size <5 cm	8.0±9.3	0.3±0.1	3.6±0.3	0.2±0.1	12.5±4.7	0.2±0.0	5.6±1.1	0.3±0.0
field size 5‐15 cm	7.9±6.3	0.2±0.1	4.8±3.8	0.2±0.1	8.6±1.5	0.2±0.1	4.5±0.0	0.2±0.1
field size >15 cm	10.0±0.8	0.2±0.0	6.2±2.0	0.2±0.1	8.6±1.4	0.2±0.0	5.9±0.0	0.1±0.1

(b)	*Agility 6 MV*			*Agility 10 MV*			*MLCi 6 MV*			*MLCi 10 MV*		
	*In‐field (%)*	*Penumbra (%)*	*Out‐of‐field (%)*	*In‐field (%)*	*Penumbra (%)*	*Out‐of‐field (%)*	*In‐field (%)*	*Penumbra (%)*	*Out‐of‐field (%)*	*In‐field (%)*	*Penumbra (%)*	*Out‐of‐field (%)*
field size <5 cm	0.6±0.4	3.0±1.3	0.4±0.2	1.6±0.8	2.7±2.1	0.7±0.2	2.8±3.8	4.2±5.3	0.6±0.2	1.9±3.9	2.5±5.0	0.4±0.2
field size 5‐15 cm	0.5±0.3	2.8±1.8	0.6±0.7	0.8±0.6	2.2±1.5	0.9±0.5	0.8±0.5	2.3±1.2	1.4±1.2	0.3±0.3	1.1±0.9	0.8±0.8
field size >15 cm	0.5±0.4	2.1±2.1	1.4±1.5	0.7±0.8	4.1±2.0	1.7±0.9	0.9±0.7	3.4±2.2	2.6±2.0	0.4±0.3	2.2±1.8	1.5±1.7
(c)	*Agility 6 MV*			*Agility 10 MV*			*MLCi 6 MV*			*MLCi 10 MV*		
	*In‐field (%)*	*Penumbra (%)*	*Out‐of‐field (%)*	*In‐field (%)*	*Penumbra (%)*	*Out‐of‐field (%)*	*In‐field (%)*	*Penumbra (%)*	*Out‐of‐field (%)*	*In‐field (%)*	*Penumbra (%)*	*Out‐of‐field (%)*
field size <5 cm	2.8±1.9	5.0±2.3	0.7±0.3	*2.2±* 0.8	5.0±1.1	*1.2±* 0.3	0.6±0.7	*1.2±* 1.6	0.4±0.2	1.1±0.7	5.3±0.8	1.0±1.1
field size 5‐15 cm	1.4±1.3	4.0±2.8	1.2±1.0	1.2±1.0	5.5±2.8	1.8±0.9	0.5±0.4	3.5±3.3	1.5±1.2	0.5±0.5	1.9±2.0	0.7±0.7
field size >15 cm	0.6±0.5	2.8±1.7	2.1±1.8	0.8±0.8	5.4±4.0	2.3±1.2	0.7±0.8	4.6±3.7	2.3±2.5	0.5±0.3	2.8±2.1	1.2±1.7

Tolerances buildup=10%; fall‐off=2%; in‐field=3%; penumbra=10%; out‐of‐field=3%.

Validation and further quantitative analysis of the agreement in the PDDs and profiles was done using independently measured data (not used in the modeling above) and applying a gamma (2% G/2 mm) acceptance criterion. PDDs for all fields were within a gamma value of 1 (pass rates for majority of points ≥ 95%), and showed good agreement, also in the buildup region. For the profiles there was generally better agreement cross‐plane compared to the in‐plane direction, where some values exceeded the gamma tolerance of 1 but were within 2, particularly for the out‐of‐field region in large field sizes and in‐field for some smaller fields.

The few results exceeding tolerance in the above analyses were accepted after careful review, based on the following: validation checks using other independently measured data, modeling constraints, magnitude of deviation from tolerance, and perceived clinical impact and significance.

There are some similarities in the physics modeling approaches for both RayStation and Pinnacle. However, a few differences pertain to the MLC modeling where the MLC offset table is used in Pinnacle whilst, in RayStation, MLC x‐position offset and MLC collimator calibration x‐position are used. Also additional interleaf leakage transmission is used in Pinnacle, whilst only average transmission is used in RayStation. The automatic modeling process in this RayStation version was occasionally found to give unpredictable results.

### B. Dosimetric tests

#### B.1 Point‐dose measurements

Results and variation of the measured output factors for open (jaw‐ and collimator‐defined) and wedged fields are shown in Table 2. The open field measurements were within 1.5% for both models, whilst the wedged fields were within 0.7% for the MLCi model and within 3% for the Agility model. Output factors for MLC only defined fields were within 1.2% for the MLCi model and within 2.6% for the Agility model.

**Figure 3:**
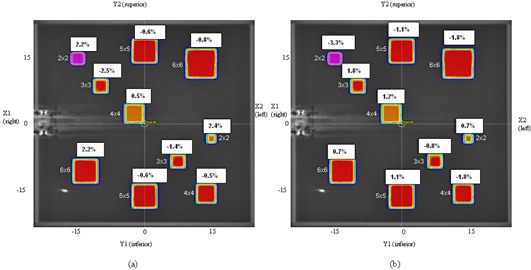
Location and results of ten off‐axis fields for Agility model, 2 × 2 cm to 6 × 6 cm measured at 10 cm depth, for point‐dose verification at (a) 6 MV and (b) 10 MV.

Most measurements were within the stated tolerances shown in Table 3 for tests using homogeneous phantoms, and the results exceeding tolerances are indicated. For central axis measurements tolerance was exceeded only for the 15° wedge measurements at 5 cm depth and field sizes beyond 25 × 25 cm, where the difference was up to 3.3%. As expected, higher off‐axis disagreements were seen at distances greater than 10 cm from the central axis. The 2 × 2 cm field size (for both MLC only and jaw‐ & MLC‐defined fields) gave the highest off‐axis differences, up to −3.3% for the Agility model at an off‐axis distance of 15 cm from the central axis (see Fig. 3). Locally there are restrictions on the minimum field aperture size allowed for 3D conformally planned off‐axis treatment plans, and generally only 6 MV is used for IMRT and VMAT planning.

**Figure 4:**
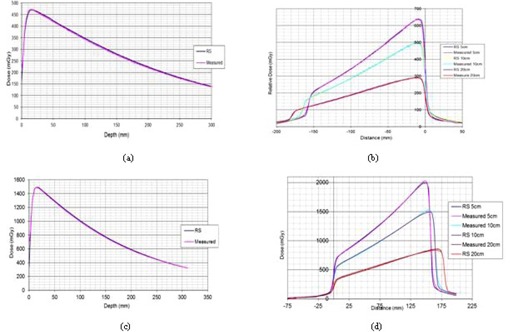
Off‐axis Agility model 15 × 15 cm wedged field: (a) depth dose in heel, (b) profiles in heel, (c) depth dose in toe, and (d) profiles in toe.

Wedged off‐axis measurements showed variation of up to 3.8% at distances > 10 cm from the central axis. Figure 4 shows an example of the measured off‐axis wedge PDDs and profiles in the heel and toe directions for a 15 × 15 cm field for the Agility 6 MV beam, measured in the center of the field and along the patient Z direction.

In addition to the above measurements, bolus of thickness ranging from 3 mm to 20 mm was applied on open and wedged fields, and the dose calculation and isodose shifts were correctly computed and displayed taking into account the presence of the bolus. Point‐dose measurements for open and wedged fields with superflab bolus in place showed a difference within −1.3% compared to the calculated dose (water‐equivalent bolus used in RayStation).

**Figure 5:**
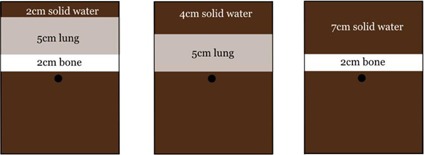
Measurement configurations for dose verification in inhomogeneous phantoms at depth of 10 cm.

For measurements using inhomogeneous slab phantoms, most results were within the stated tolerances shown in Table 4 for the geometrical configurations shown in Fig. 5. The tolerance was exceeded for the 6 MV MLCi beam with the bone insert, and the trend for this energy and depth was generally lower dose predicted by the RayStation model. The point doses for the more challenging inhomogeneous geometry showed excellent agreement, with composite doses being within ± 1% for both 6 MV Agility beam models from both TPS (see Table 5).

**Table 2:** Variation in output factors, showing average percentage difference and standard deviation (± 2 SD) for field sizes from 1 × 1 cm to 40 × 40 cm open, and 3 × 3 cm to 40 × 30 cm wedged, normalized to a 10 × 10 cm field, 100 cm SSD, and 10 cm depth

	*Agility 6 MV*		*Agility 10 MV*		*MLCi 6 MV*		*MLCi 10 MV*	
	*Open Fields*	*Wedged Fields*	*Open Fields*	*Wedged Fields*	*Open Fields*	*Wedged Fields*	*Open Fields*	*Wedged Fields*
field size <5 cm	‐0.4±1.8	0.4±0.1	‐0.4±2.8	‐0.4±0.1	‐0.3±1.4	0.3±0.0	‐0.5±0.3	0.4±0.1
field size 5‐15 cm	0.0±0.0	0.0±0.2	0.0±0.6	0.6±2.5	0.1±0.2	0.3±0.1	‐0.1±0.4	0.0±0.2
field size >15 cm	0.6±0.9	0.6±0.8	0.6±1.0	0.0±0.0	0.2±0.4	0.5±0.4	‐0.2±0.4	0.5±0.5

Tolerances open=2%; wedged=3%.

**Table 3:** Variation of point doses in homogeneous medium, RayStation vs. measured, showing maximum to minimum differences for checks performed at 5, 10, and 20 cm depths for open and wedged fields, for field sizes ranging from 1 × 1 cm to 40 × 40 cm (unless stated)

				*Range of Difference (%)*				
*Medium*	*Test*		*Depth*	*Agility 6 MV*	*Agility 10 MV*	*MLCi 6 MV*	*MLCi 10 MV*	*Tolerance*
Homogeneous (water & plastic phantoms)	Open central axis point doses		5 cm	1.8% to −0.7%	0.8% to 0.1%	0.6% to 0.1%	1.7% to −0.5%	2%
			10 & 20 cm	1.7% to −1.1%	1.8% to −0.1%	0.3% to −0.4%	1.3% to −1.0%	
	Wedged central axis point doses	W15	5 cm	3.3% to 2.5%^a^	1.5% to 0.8%	0.1% to −1.3%	1.7% to 1.3%	3%
			10 & 20 cm	2.5% to 0.8%	1.7% to 0.9%	0.9% to −1.5%	1.6% to 1.4%	
		W30	5 cm	2.8% to 1.9%	1.1% to 0.4%	1.2% to −0.2%	1.8% to 1.5%	
			10 & 20 cm	2.4% to 1.1%	1.6% to 0.5%	0.2% to −1.6%	2.0% to 1.4%	
		W45	5 cm	1.9% to −0.9%	1.0% to −0.2%	‐0.2% to ‐1.1%	2.8% to 2.0%	
			10 & 20 cm	1.7% to 0.0%	1.4% to 0.2%	‐0.9% to ‐1.8%	2.7% to 2.1%	
		W60	5 cm	1.0% to −1.7%	1.0% to −1.7%	0.8% to −1.6%	2.6% to 2.2%	
			10 & 20 cm	1.3% to −1.9%	1.3% to −1.9%	0.3% to −1.1%	2.8% to 2.1%	
Homogeneous (water & plastic phantoms)	Open off‐axis point doses (see Fig. 3 for distances off‐axis)	>10cm from CAX	5 cm	1.0% to −0.1%	1.1% to −0.6%	1.0% to −0.1%	1.3% to −0.6%	3%
			10 cm	0.5% to −2.5%	1.8% to −0.8%	0.5% to −2.5%	1.0% to −2.4%	
		≥ 10 cm from CAX	5 cm	3.3% to −0.1%*	1.7% to −3.2%*	2.4% to −0.1%	2.1% to −1.4%	
			10 cm	2.4% to −0.8%	1.8% to −3.3%*	2.7% to −0.8%	0.8% to −2.1%	
	Wedged central off‐axis point doses (W15, W30, W45,W60)	Toe	5, 10, & 20 cm	3.8% to 2.1%	1.3% to −4.0%	3.0% to 0.5%	3.6% to 1.7%	4%
		Heel	5, 10, & 20 cm	2.7% to −1.5%	1.5% to −3.2%	3.9% to −0.8%	4.4% to −1.7%^a^	
Homogeneous (water & plastic phantoms)	Extended SSDs central axis (5×5 cm, 10×10 cm, 5×40 cm fields)	80 cm	5 cm		1.2% to‐0.4%^b^			2%
		SSD	10 cm		1.5% to −0.8%^b^			
		100 cm	5 cm		0.8% to −1.0%^b^			
		SSD	10 cm		1.0% to −1.8%^b^			
		120 cm	5 cm		0.4% to −1.6%^b^			
		SSD	10 cm		0.5% to −2.4%^b^			
Homogeneous	Oblique incidence (10×10 cm)	G=45°	5, 10, 20 cm		1.2% to −1.4%^b^			2%

a Tolerance exceeded.

b Range across all machines.

CAX = central axis.

**Table 4:** Variation of point doses in inhomogeneous medium, RayStation vs. measured, performed at 10 cm depth for open (field sizes from 3× 3 cm to 40× 40 cm) and wedged fields (field sizes from 3 × 3 cm to 30× 40 cm)

		*Range of Difference (%)*				
*Medium*	*Test*	*Agility 6 MV*	*Agility 10 MV*	*MLCi 6 MV*	*MLCi 10 MV*	*Tolerance*
Inhomogeneous ‐ lung	Open central axis point doses	0.1% to −0.8%	1.6% to 0.3%	0.1% to −0.8%	0.2% to 0.9%	3%
	Wedged central axis point doses (W60)	1.6% to −1.2%	1.0% to −0.9%	‐0.1% to ‐0.9%	0.8% to −0.5%	4%
Inhomogeneous ‐ bone	Open central axis point doses	‐1.0% to ‐2.7%	0.3% to −0.6%	‐1.6% to ‐3.1% ^a^	1.7% to −1.8%	3%
	Wedged central axis point doses (W60)	0.0% to −3.1%	‐0.9% to ‐2.5%	0.9% to −3.4%^a^	‐1.2% to ‐2.1%	4%
Inhomogeneous ‐ bone and lung	Open central axis point doses	1.8% to −1.5%	1.3% to −0.2%	‐1.7% to ‐3.1% ^a^	0.0% to −2.6%	3%
	Wedged central axis point doses (W60)	1.3% to −1.9%	‐0.3% to −1.8%	0.7% to −3.5%^a^	2.1% to −2.2%	4%

a Tolerance exceeded.

**Table 5:** Summary of point‐dose results for the 6 MV Agility beam models in RayStation and Pinnacle for the anthropomorphic thorax phantom

		*Mean & Range of Measurements*		
*Medium*	*Test*	*RayStation Agility 6 MV*	*Pinnacle Agility 6 MV*	*Tolerance*
Thorax Phantom	Open CAX doses	−0.6%(2.4% to−3.2%)	0.4% (2.7% to−2.5%)	3%

#### B.2 MLC film checks

There was generally a good match between the film measurements and the RayStation MLC models, mostly within 1.5% dose difference relative to the maximum, taking into account the inherent uncertainties with film dosimetry. Picket Fence and Garden Fence tests were used to confirm accuracy of the MLC transmission modeling and primary source size. Picket Fence results correctly showed the expected match for seven abutting field segments, and also accurately reproduced the 1 mm and 2 mm gaps and overlaps from the planning system.

**Figure 6:**
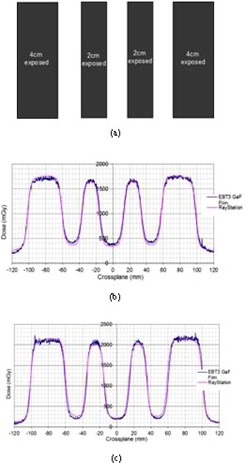
Simplified Garden Fence field setup for 4 cm and 2 cm exposed areas and 2.5 cm shielded areas (a), and results for MLCi model (b) and Agility model (c).

The Garden Fence (step and shoot) test setup and results are shown in Fig. 6. It was observed in this case that, for the MLCi model, the film measurement marginally underestimated the exposed area, whilst overestimating the shielded areas (less than 25 mGy difference in both cases). For the Agility model, the film measurement marginally underestimated the dose in the shielded areas. The model values used for transmission were aimed at getting as good a fit in‐plane as well as cross‐plane. For the Agility model, the MLC transmission value of 0.5% was used for 6 MV and 1% for 10 MV, whilst for the MLCi model, values of 2% were used for both 6 and 10 MV.

**Figure 7:**
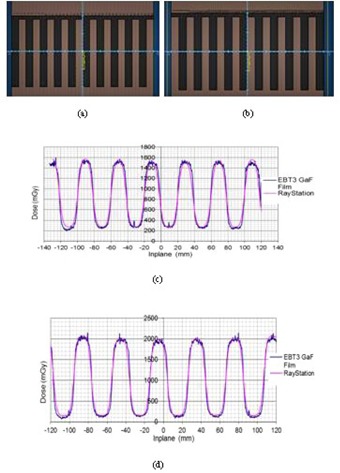
Fingers test field setup for (a) MLCi model and (b) Agility model, and the corresponding results (c) and (d), showing good match to EBT3 GAFCHROMIC film.

For the Fingers tests, based upon scans across static MLCs to verify the tongue‐and‐groove modeling, typical scans across MLCi and Agility leaves in Fig. 7 show acceptable agreement. Note that there is no physical tongue and groove for the Agility collimator; however, modeling parameters from both models can be adjusted during modeling.

**Figure 8:**
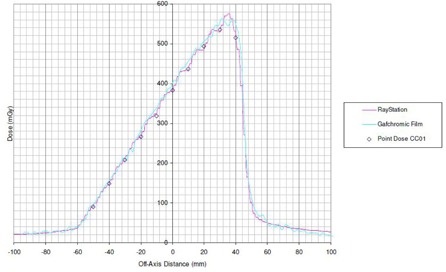
Strip test pattern result of ten (1 cm × 10 cm) step‐and‐shoot segments for the Agility model, showing RayStation profile (1 mm resolution), measured film profile, and CC01 point dose.

For the Strip Pattern test, used to validate MLC position offset and transmission, and consisting of 10 adjacent (1 cm × 10 cm) strips planned using step and shoot for the Agility model, comparisons to film and CC01 point doses are shown in Fig. 8, all measurements at 10 cm depth, 90 cm SSD. The CC01 showed excellent agreement to the TPS, whilst the film marginally overestimated the dose in the high‐dose region.

#### B.4 Clinical plans

##### B.4.1 Standard clinical cases

**Figure 9:**
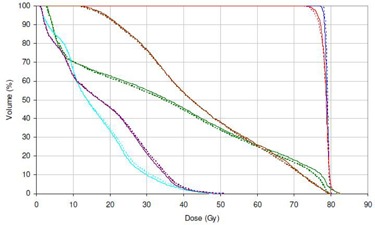
Typical DVH comparison for prostate VMAT plan showing 3DVH (solid line) and RayStation (dotted line) results, prescription (Px) dose 78 Gy. Blue=CTV,red=PTV,green=bladder,brown=rectum,turquoise=left femur,violet=right femur.

For the prostate IMRT and VMAT (including dual arc) plans, the mean point‐dose difference was 0.3% ± 1.2%, whilst the mean gamma (3%G/3 mm) was 99.2% ± 1.5%, mean gamma (2%G/2 mm) was 94.2% ± 3.2% and mean gamma (2%L/2 mm) was 85.3% ± 7.5%. Comparisons to 3DVH were also performed for the prostate plans — Fig. 9 shows a typical example at 2%G/2 mm. PTV D95% (dose to 95% of the volume) and $D_{\text{mean}}$ differences were less than 1% between 3DVH and RayStation, whilst all OAR point of interest dose differences were less than 4%. There were no unexpected biases or systematic errors detected in the visual dose grid assessments or in the dose profile matches. CC13 in‐phantom measured point doses for other treatment sites, including five brain and five breast plans, and the palliative cases were all within 2% of the RayStation doses.

##### B.4.2 Complex clinical cases

**Figure 10:**
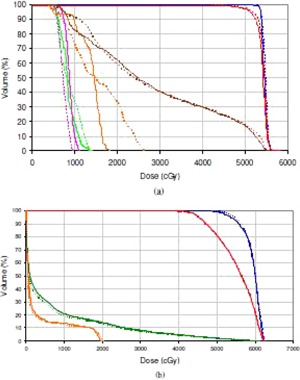
DVH comparison for (a) H&N VMAT plans, Px 54 Gy, and (b) noncoplanar hypofractionated left lung forward‐planned IMRT treatment plans, Px 50 Gy, from Pinnacle (solid line) and RayStation (dotted line). Blue=CTV,red=PTV,bright green=left parotid,pink=brainstem,brown=mandible,orange=spinal cord,green=left lung.

Comparison of the DVHs between RayStation and Pinnacle for the complex H&N and lung cases is shown in Fig. 10. Similar agreement in DVHs was observed for the stereotactic spine case. For all cases equivalent or slightly better CTV and PTV dose coverage was attained with RayStation, whilst in some instances lower OAR doses were obtained compared to Pinnacle.

**Figure 11:**
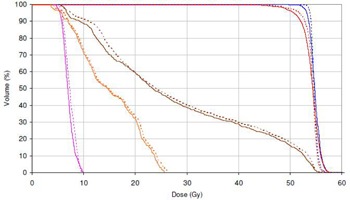
DVH comparison for H&N VMAT plans from 3DVH (solid line) and RayStation (dotted line). Blue=CTV,red=PTV,orange=spinal cord,pink=brainstem,brown=mandible.

DVH analysis performed for RayStation against 3DVH is shown in Fig. 11 for the example of the H&N case at 2%G/2 mm. The 3DVH comparisons in all complex cases showed PTV D95% and $D_{\text{mean}}$ dose differences of less than 1% against RayStation, whilst all OAR point of interest doses were within 3% difference and remained below acceptable tolerances. In addition, inspection of the TPS dose grid and the resultant metrics against 3DVH was also conducted and results were satisfactory in all cases, whilst dose profiles through regions of interest showed no areas of significant underdosage or overdosage. The point‐dose differences to ionization chamber measurements and gamma indices for each complex plan (Table 6) show good correlation of the metrics for RayStation and Pinnacle and acceptable pass rates for the point doses, gamma indices at 3%G/3 mm, and 2%G/2 mm, whilst results obtained at 2%L/2 mm are comparable to other studies,^12^, ^13^ for complex clinical cases.

**Table 6:** Complex clinical cases QA results showing composite point‐dose measurement difference and gamma pass rates at 10% dose threshold, with measurement uncertainty applied

		*Point Dose % diff. (TPS‐Measurement)*	*Gamma (absolute dose) Values*		
*Case*	*TPS*		3%G/3 mm	2%G/2 mm	2%L/2 mm
SRT Spine (IMRT)	RayStation	0.7%	96.1	93.3	85.1
	Pinnacle	−1.1%	98.8	95.7	86.3
Hypofractionated Lung (forward‐planned IMRT)	RayStation	0.5%	98.3	94.9	85.3
	Pinnacle	−1.3%	100	96.1	84.6
H&N (dual‐arc VMAT)	RayStation	−1.4%	96.6	90.1	83.6
	Pinnacle	0.8%	95.8	91.6	81.3

#### B.5 Plan assessment tools

A small difference was observed in the volume calculated in the DVH statistics and that calculated in the ROI properties. This difference is due to different discretizations (i.e., in the DVH dose statistics, the volume is calculated on the dose grid and, in ROI properties, the volume is calculated on a different grid having a resolution close to the CT resolution) (B. Mzenda, private correspondence with RaySearch, June 4, 2013). Studies of the different plan pairs between Pinnacle and RayStation, including dose grid scrutiny and analysis of critical DVH points and comparisons to 3DVH, show that this difference has no significant effect on the TPS accuracy and calculated plan metrics.

## IV. DISCUSSION

### A. Beam modeling

Acceptable beam models were attained for the Agility and MLCi machines, based on the match to measured data. Modeling limitations prevented the ability to match correspondingly well over all field sizes, particularly for the tails and shoulders, and a better match at smaller field sizes was prioritized over that at larger field sizes to reflect the clinical balance (majority of treatments VMAT/IMRT based) at our center. Optimizing for smaller field sizes and compromising larger field sizes is also common and acceptable for most treatment planning systems. The trend that the in‐field values have higher RMS errors, as shown in Table 1, is partly related to the definition of the in‐field region which starts at the 80% penumbra line. This choice might not be the best, and separating the TPS reported profile regions into tail, heel, and shoulder areas would be useful for analysis.

As shown in the results, it was not always possible to get good agreement in the out‐of‐field regions, particularly for larger field sizes. A possible explanation of this could be the use of the no‐tilt kernel approximation in RayStation. Whilst this is satisfactory for most clinical cases and saves computation time, is can also lead to dose errors outside the field edge, as well as off‐axis. Another possibility for this could be the lack of a separate energy spectrum, TERMA, and dose tracing for the flattening filter source. The kernel tilt error is partly corrected for via inverse square law TERMA and dose rescaling (RayStation 3.5 Reference Manual 2013, p.65^(14)^). The effect of out‐of‐field regions stacked on top of each other in multiple‐segment IMRT fields was evaluated using clinical test plans.

The modeling in RayStation currently does not support the direct import of published beam energy spectra, so modeling is based on the beam spectrum for the matching machine type for which the energy fluence and electron contamination can be adjusted, as required. The beam spectrum can be manually adjusted to attain a corresponding published spectrum, if required. For the buildup region, it was particularly difficult to obtain a match to measured data in RayPhysics modeling, especially for the larger fields in the Agility model and the smaller fields in the MLCi model, where this difference exceeded 10% in a couple of instances. However, this discrepancy was significantly less in the model validation comparison to measured data acquired using different detectors and FSDs. It is, therefore, possible that this may be due to measurement variations. However, the RayStation electron contamination model is restricted by a two source fluence computation and a two parameter energy spectrum, and is not allowed to adapt freely to the buildup (B. Mzenda, private correspondence with RaySearch, 10 February, 2013).

Manual modeling was found to work better than automodeling, which occasionally resulted in unpredictable results. MLC‐ and jaw‐collimated rectangular fields were used in the modeling; hence, third‐party software (OmniPro ImRT v7.1 (IBA Dosimetry) and Excel) was needed to tune the MLC parameters based on the match from the beam 3D modeling dose profiles to measured test fields. This has implications on the commissioning time due to the additional steps involved, as well as the hidden cost of the additional software to that of the planning system. Other observations include the limitation on the exported file size accepted by the third‐party software balanced against the requirement to get adequate resolution for analysis.

### B. Dosimetric tests

Open field central axis point doses were all within 2% (including extended and near SSD) and generally within 3% for central axis wedged fields (maximum deviation of 3.3% for a 25 × 25 cm and 30 × 30 cm 15° wedged field, 6 MV). Off‐axis open and wedged fields and doses in homogeneous and inhomogeneous mediums were mostly within 4%, whilst oblique field doses were within 1.5%. An error was identified in this version of the software for the Elekta motorized wedge, whereby the modified energy spectrum was used for both the wedged and open parts of the wedged field;^(15)^ however, using the suggested workaround resulted in an improvement in the agreement for the wedged fields.

The composite doses for the anthropomorphic phantom were within ± 1.0% for the 6 MV Agility beam model from both TPS (see Table 5). These results demonstrate excellent agreement given the size of the fields and the nature of the phantom, being consistent with previous published results of the CCC algorithm.^16^, ^17^ Larger errors were evident for some beam geometries that can be attributed to the variation in equivalent path length across the field width at the measurement point that would increase the sensitivity of the chamber placement. This effect was most prevalent for those beams glancing the phantom surface, or those that passed through other irregularities (e.g., heart). These errors can also be attributed to the uncertainty in the dose calculation in close proximity to the lung/tissue interface in the presence of lateral electron disequilibrium, that is expected to reduce as the field and tumor size increases for a given energy.^(18)^

Good agreement was seen between the manually calculated and RayStation volumes. The difference in margin growth between RayStation and Pinnacle is mainly due to different techniques used by these planning systems for volume expansion. In RayStation, for uniform margins the Fast Marching Distance transform is used^(19)^ and, for nonuniform margins, a chamfer‐based approach is used.^(20)^ On the other hand, Pinnacle uses a voxel summation technique, where the edge voxels are half‐weighted.^(21)^

Plan quality corresponding to Pinnacle TPS has been attained using the new models for the clinical cases used in this study. There was also good correlation in specific DVH metrics from RayStation and those from the perturbed 3DVH dose distributions. For this plan quality assessment of several pairs of clinical plans historical and more sensitive metrics were applied, such as the 2%G/2 mm and 2%L/2 mm criteria. This was particularly in order to visually study dose difference patterns and profiles to deduce any possible systematic errors. Dose verification, using a small volume ionization chamber and the ArcCHECK device for the small segment IMRT and VMAT plans, showed absolute dose differences of less than 2%.

## V. CONCLUSIONS

A systematic beam modeling process and rigorous dosimetric evaluation, applying sensitive dose metrics, suitably chosen measurement systems, and tighter tolerances, has been applied to the RayStation TPS. Demonstrably equivalent plan quality to Pinnacle has been attained, whilst point‐dose differences, gamma indices, and 3D dose correlations to 3DVH show no noticeable systematic dosimetric errors. The RayStation TPS has been tested in challenging geometries, as well as in some complex clinical applications, and its overall performance has been found to be satisfactory.

## ACKNOWLEDGMENTS

The authors would like to thank RaySearch Laboratories for their assistance during the commissioning and clinical implementation phases of this project, as well as for discussions pertaining to the modeling and other physics issues relating to the TPS. Special thanks also to the planning Radiation Therapists' team at Auckland Radiation Oncology for optimization of the different treatment plans used in this study, and to Laura Ciurlionis for the physics contributions to this study.

## Supporting information

Supplementary Material

Supplementary Material

Supplementary Material

Supplementary Material

Supplementary Material

Supplementary Material

Supplementary Material
