# Supplementary figures and images for: Modeling and dosimetric performance evaluation of the RayStation treatment planning system
Source: J Appl Clin Med Phys. 2014 Sep 8;15(5):29–46. doi: 10.1120/jacmp.v15i5.4787 (PMC5711080; doi:10.1120/jacmp.v15i5.4787)

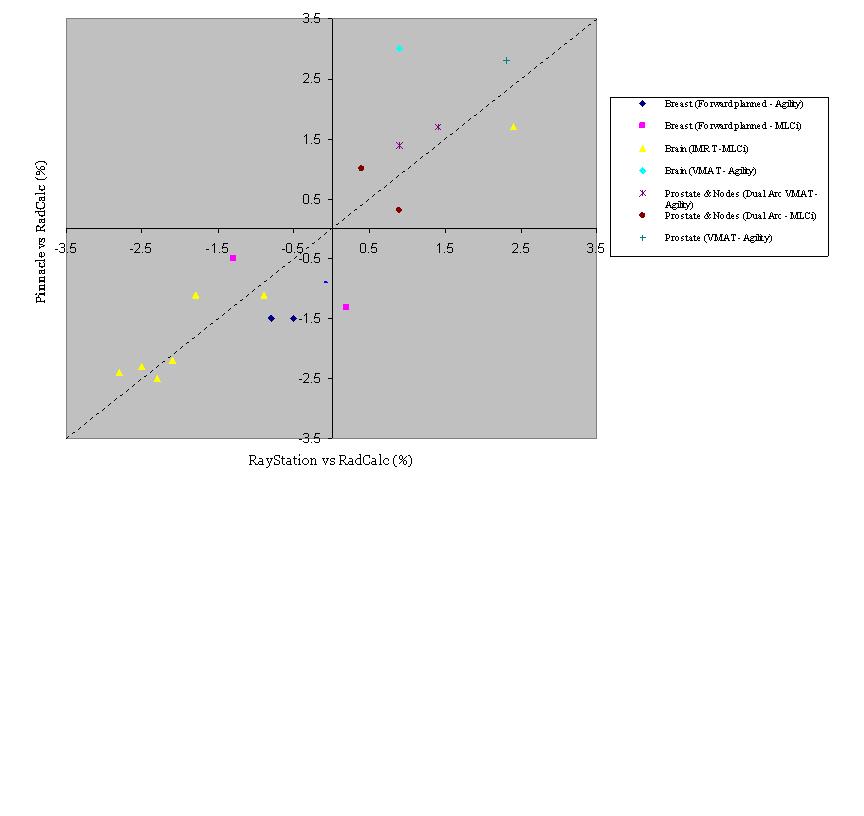

Supplement: Supplementary file 1 — Supplementary Material [file ACM2-15-029-s001.JPG]

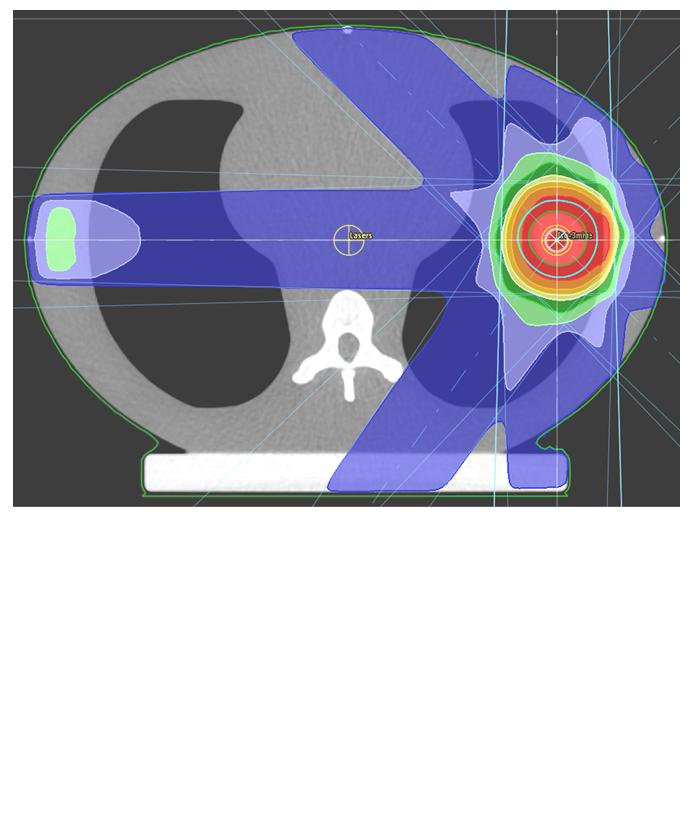

Supplement: Supplementary file 2 — Supplementary Material [file ACM2-15-029-s002.JPG]

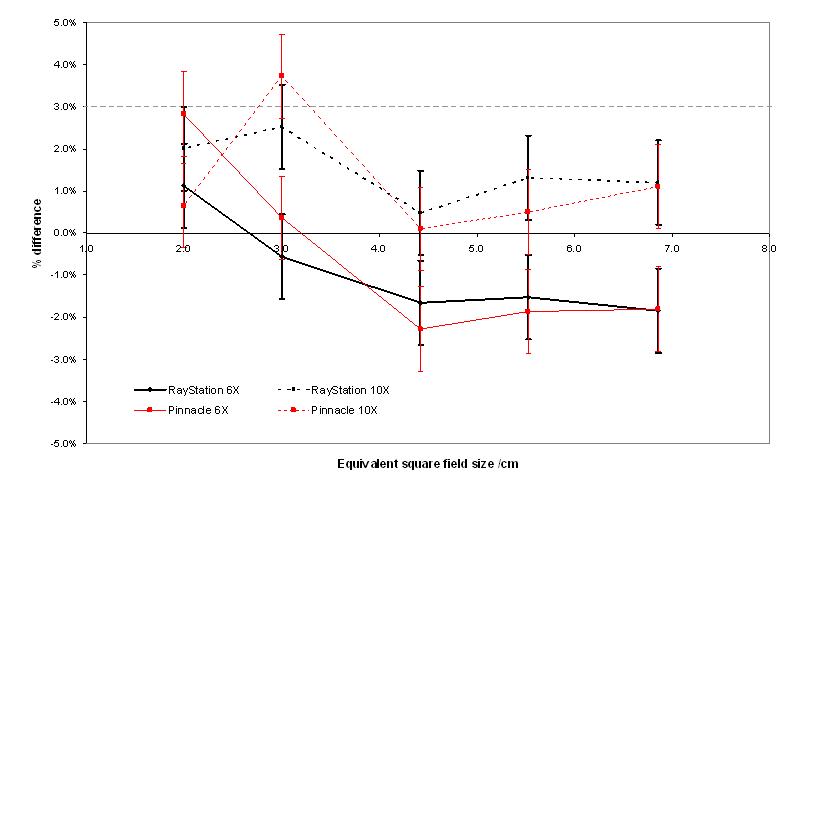

Supplement: Supplementary file 3 — Supplementary Material [file ACM2-15-029-s003.JPG]

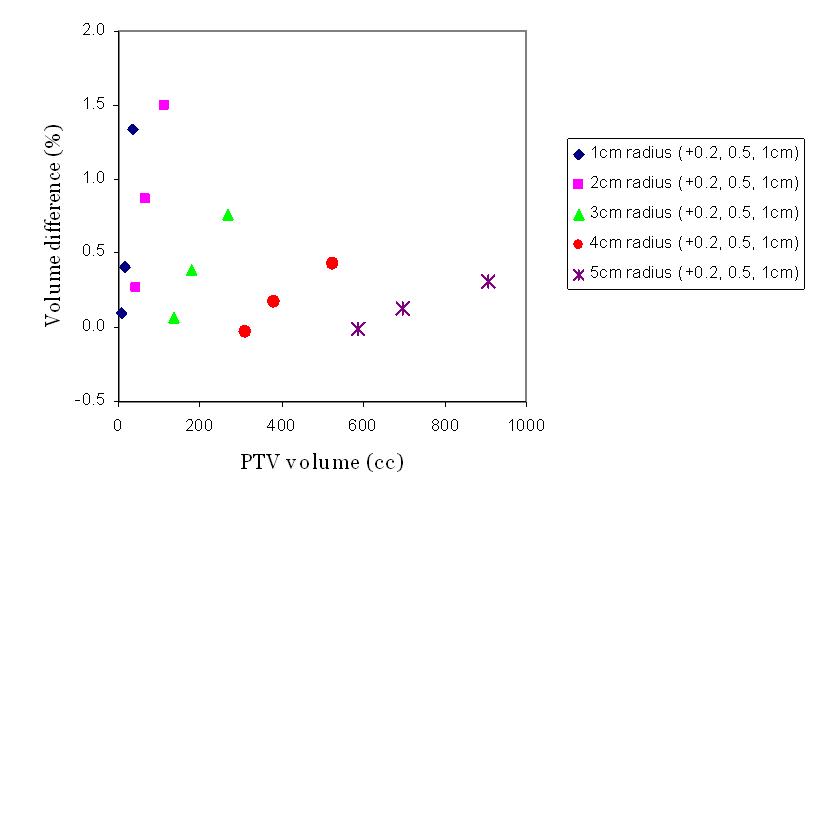

Supplement: Supplementary file 4 — Supplementary Material [file ACM2-15-029-s004.JPG]

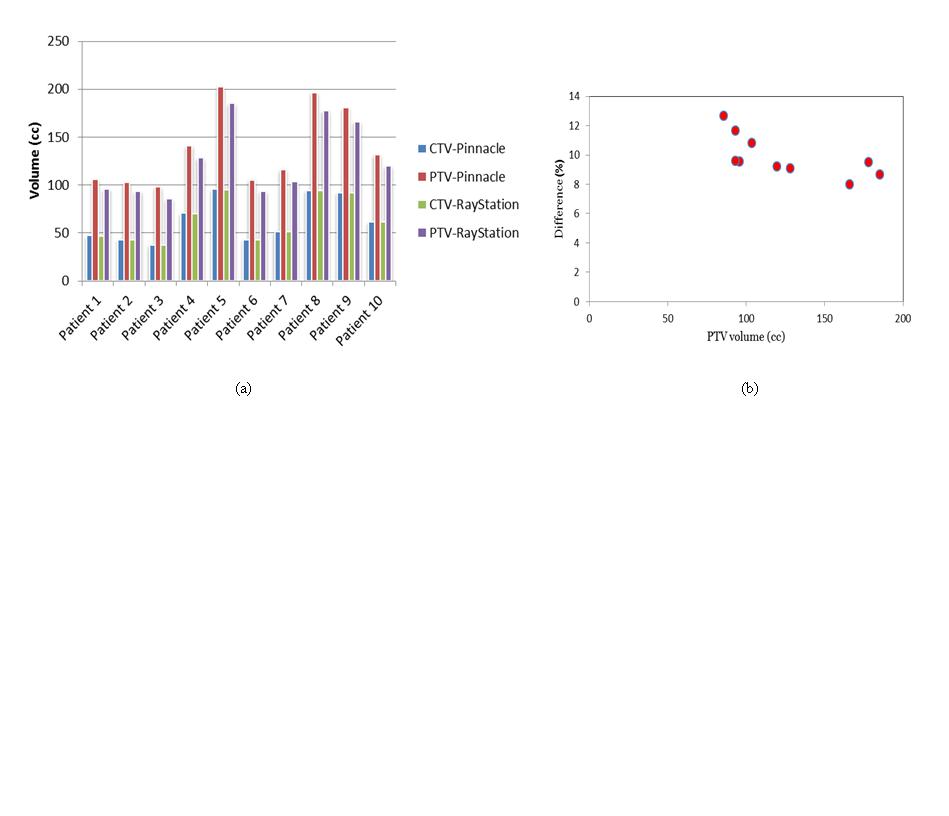

Supplement: Supplementary file 5 — Supplementary Material [file ACM2-15-029-s005.JPG]

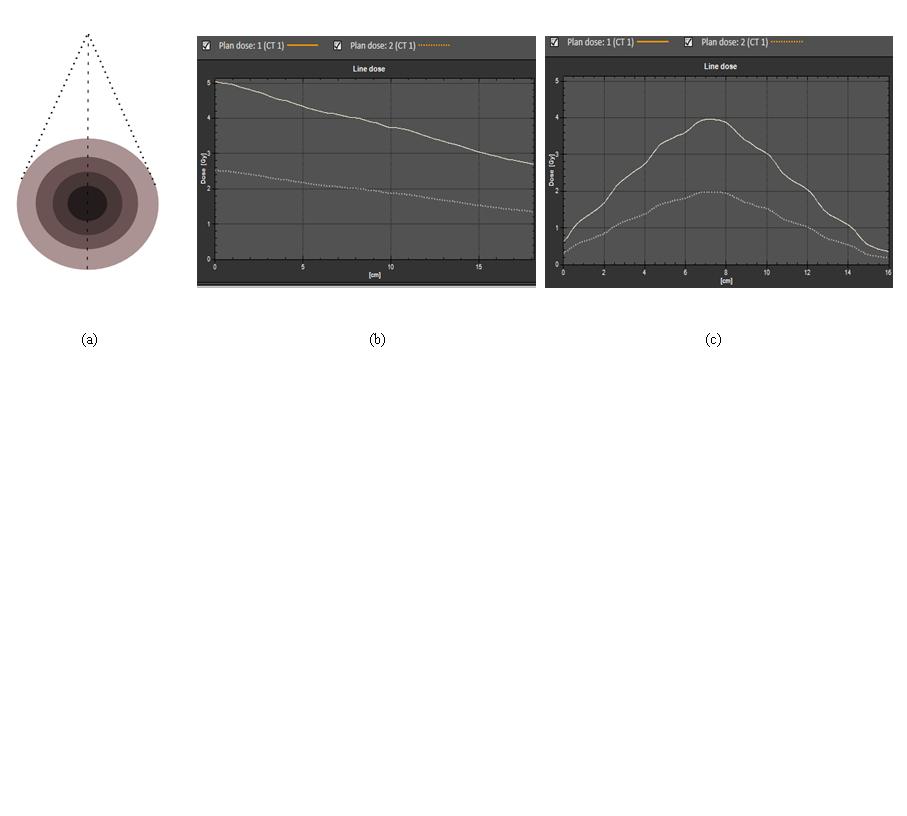

Supplement: Supplementary file 6 — Supplementary Material [file ACM2-15-029-s006.JPG]

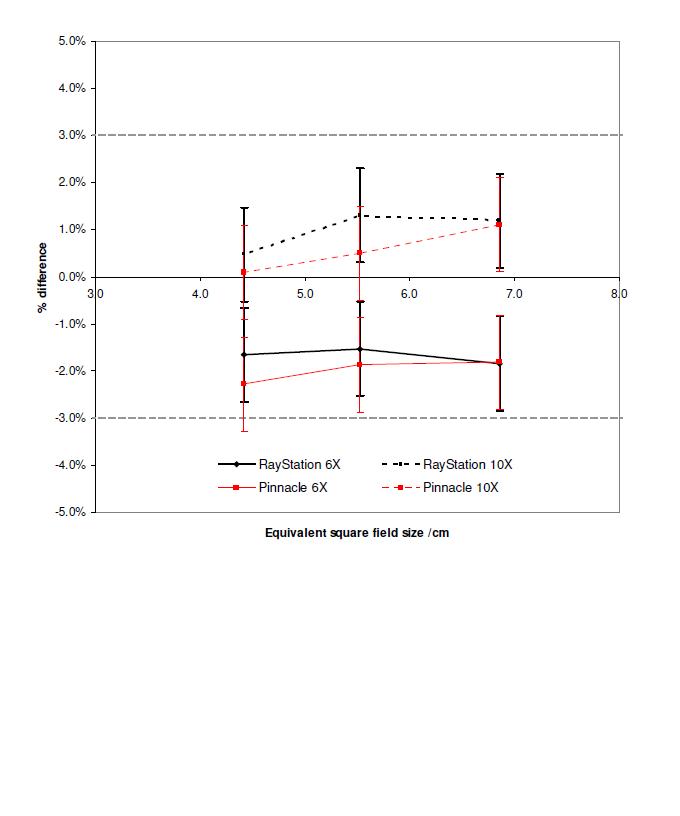

Supplement: Supplementary file 7 — Supplementary Material [file ACM2-15-029-s007.JPG]
